# Reductive enzymatic dynamic kinetic resolution affording 115 g/L (*S*)-2-phenylpropanol

**DOI:** 10.1186/s12896-021-00715-5

**Published:** 2021-10-11

**Authors:** Christian Rapp, Simone Pival-Marko, Erika Tassano, Bernd Nidetzky, Regina Kratzer

**Affiliations:** 1 Institute of Biotechnology and Biochemical Engineering, Graz University of Technology, NAWI Graz, 8010 Graz, Austria; 2 Department of Chemistry, University of Graz, NAWI Graz, Heinrichstrasse 28, 8010 Graz, Austria; 3 Austrian Centre of Industrial Biotechnology (ACIB), 8010 Graz, Austria

**Keywords:** Enantiopure 2-aryl-1-propanol, Reductive dynamic kinetic resolution, Biocatalyst stability, Aldo–keto reductase engineering

## Abstract

**Background:**

Published biocatalytic routes for accessing enantiopure 2-phenylpropanol using oxidoreductases afforded maximal product titers of only 80 mM. Enzyme deactivation was identified as the major limitation and was attributed to adduct formation of the aldehyde substrate with amino acid residues of the reductase.

**Results:**

A single point mutant of *Candida tenuis* xylose reductase (*Ct*XR D51A) with very high catalytic efficiency (43·10^3^ s^−1^ M^−1^) for (*S*)-2-phenylpropanal was found. The enzyme showed high enantioselectivity for the (*S*)-enantiomer but was deactivated by 0.5 mM substrate within 2 h. A whole-cell biocatalyst expressing the engineered reductase and a yeast formate dehydrogenase for NADH-recycling provided substantial stabilization of the reductase. The relatively slow in situ racemization of 2-phenylpropanal and the still limited biocatalyst stability required a subtle adjustment of the substrate-to-catalyst ratio. A value of 3.4 g_substrate_/g_cell-dry-weight_ was selected as a suitable compromise between product *ee* and the conversion ratio. A catalyst loading of 40 g_cell-dry-weight_ was used to convert 1 M racemic 2-phenylpropanal into 843 mM (115 g/L) (*S*)-phenylpropanol with 93.1% *ee*.

**Conclusion:**

The current industrial production of profenols mainly relies on hydrolases. The bioreduction route established here represents an alternative method for the production of profenols that is competitive with hydrolase-catalyzed kinetic resolutions.

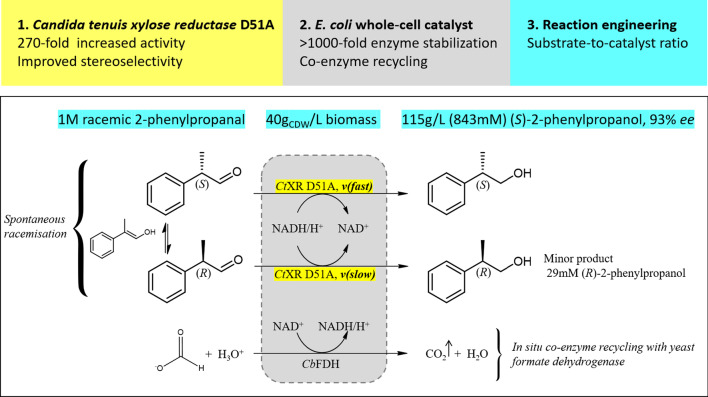

**Supplementary Information:**

The online version contains supplementary material available at 10.1186/s12896-021-00715-5.

## Highlights

● The D51A mutant of *Candida tenuis* xylose reductase showed a 270-fold higher enzymatic activity and improved enantioselectivity for (*S*)-2-phenylpropanal.

● Use of a whole-cell catalyst stabilized the enzyme > 1000-fold under reaction conditions.

● Efficient kinetic resolution of racemic 2-phenylpropanal by the whole-cell catalyst was demonstrated.

● (*S*)-2-phenylpropanol was produced with a titer of 843 mM (115 g/L) and 93.1% *ee.*

● The substrate-to-biocatalyst ratio was the main factor determining the enantiopurity and final titer of the product.

## Background

**Fig. 1:**
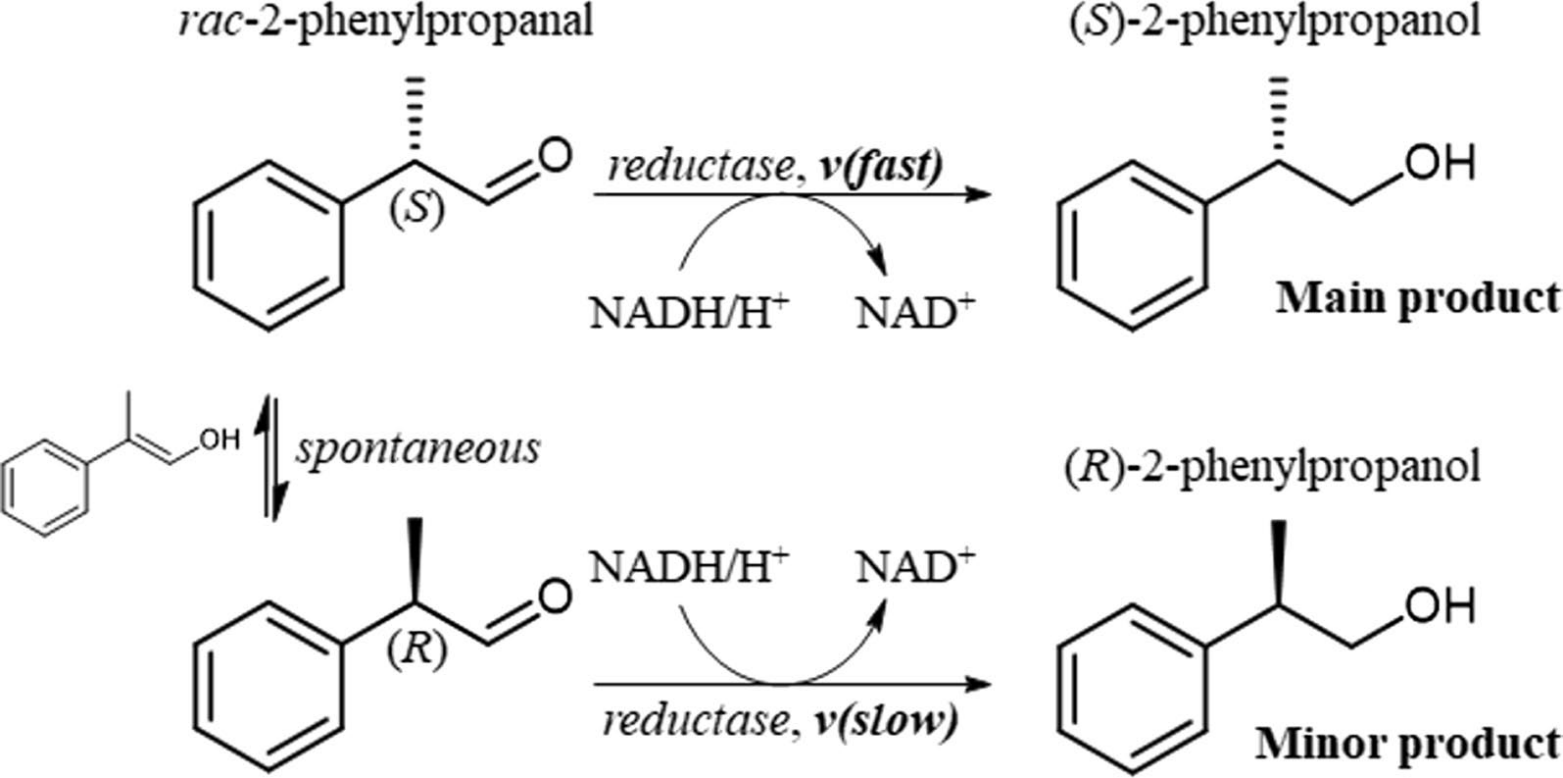
Reductive enzymatic dynamic kinetic resolution of racemic 2-phenylpropanal

2-Aryl-1-propanols are crucial synthons of profen-type non-steroidal anti-inflammatory drugs (NSAIDs) [1]. The simplest representative of this class, 2-phenylpropanol, has an odor resembling lilac or hyacinth, and is used as a fragrance ingredient in personal care products, as well as a precursor in the synthesis of further fragrances [2, 3]. The different biological activities and odors of *R*- and *S*-profens have inspired studies on the production of optically pure 2-aryl-1-propanols. A number of biocatalytic routes have been proposed over the years, including kinetic resolutions by hydrolases, nitrile-converting enzymes or oxidases, asymmetrization of prochiral precursors through enzymatic decarboxylation, and isomerization by styrene oxide isomerase. Kinetic resolutions using hydrolases and oxidoreductases are the two most mature strategies (reviewed in [4]). The economic attractiveness of kinetic resolutions, which are restricted to maximally 50% yield, can be increased by in situ racemization of the unused antipode, resulting in dynamic kinetic resolutions (DKR). DKR strategies exploiting hydrolases and oxidoreductases make use of the relatively fast racemization of 2-aryl-1-propanoic acids/esters and 2-aryl-1-propanals (Fig. 1). Hydrolase-catalyzed kinetic resolutions were the first biocatalytic routes towards enantiopure profens, and numerous lipases and esterases have been tested for their enantioselectivities towards several profens [5]. Product concentrations of 0.5 M and enantiopurities of up to 99% *ee* were reported, leading to the application of hydrolases at the industrial scale [6, 7]. Although hydrolases remain attractive in terms of their process performance, they offer limited opportunities to valorize intellectual property [4]. Oxidoreductases are an interesting alternative, but the published biocatalytic routes for accessing chiral 2-aryl-1-propanols using oxidoreductases show markedly lower product concentrations of maximally 80 mM [4, 8–17]. Here, we report a reductive enzymatic dynamic kinetic resolution for the preparation of (*S*)-2-phenylpropanol. The xylose reductase from *Candida tenuis* (*Ct*XR, aldo–keto reductase superfamily) showed a basal activity with *rac*-2-phenylpropanal and moderate preference for the (*S*)-enantiomer. We investigated five enzyme variants with single point mutations in close vicinity to the stereocenter of 2-phenylpropanal. The D51A mutant of *Ct*XR showed high catalytic activity with excellent enantioselectivity for (*S*)-2-phenylpropanal, and was integrated into a reductive whole-cell catalyst based on *E. coli*. Reaction optimization resulted in high enantioselectivity and product concentration at full conversion. The reductive enzymatic dynamic kinetic resolution established in this study is comparable with lipase-based processes in terms of product concentration, offering a viable alternative for industrial applications.

## Results

*Ct*XR, which converts xylose to xylitol in the central sugar metabolism of *Candida tenuis*, is a member of the aldo–keto reductase (AKR) superfamily. Like many other AKRs, *Ct*XR has broad substrate specificity, and several substrate-binding site mutants with improved specificity for aromatic ketones were used in the synthesis of (*R*)-ethyl mandelates and (*S*)-phenylethanols [18, 19]. However, the application of *Ct*XR as an industrial biocatalyst was hindered by its moderate catalytic activity and low stability. Motivated by a moderate intrinsic activity of wild-type *Ct*XR on *rac*-2-phenylpropanal (*k*_cat_/*K*_m,rac_ 130 s^−1^ M^−1^, *k*_cat_ 0.05 s^−1^), we tested a number of single point mutations targeting the substrate-binding site for their effects on the activity and enantioselectivity of the enzyme in the reduction of 2-phenylpropanal.

### CtXR mutants

The substrate-binding cavity of aldo–keto reductases is mainly formed by residues from three large and flexible loops [20, 21]. Loop flexibility provides the structural basis for the relaxed substrate specificity, but complicates rational engineering [22]. A model of the binding mode of the natural substrate d-xylose (open chain form) indicated that the C-1 of xylose is positioned within hydride-transfer distance above the C-4 of nicotinamide, with a hydrogen bond between the carbonyl oxygen and the general acid catalyst Tyr-52. In this model, the aldehyde hydrogen was oriented towards the indole ring of Trp-24 and the C-2 hydroxyl interacted with Asn-310, while the hydroxyl groups of C3, C4 and C5 interacted with Asp-51 [23]. Here, we probed wild-type *Ct*XR and individual point mutants of the main substrate recognition residues Trp-24, Asn-310 and Asp-51 as catalysts for the reduction of 2-phenylpropanal. The replacement of Trp-24 with smaller phenylalanine and tyrosine was previously shown to increase enzyme activity on bulky ketone substrates [18, 24]. Interaction between the Nδ1 of Asn-310 and the C-2(*R*) hydroxyl of sugars is considered the basis for substrate discrimination in *Ct*XR [23, 24]. Asp-51 accounts for a major part of the relative polarity of the binding site, and its substitution with alanine led to improved activity with the aromatic ketone *o*-chloroacetophenone [19].

### Reduction of 2-phenylpropanal by *Ct*XR variants

Table 1 summarizes the steady-state kinetics of NADH-dependent reduction of racemic and (*S*)*-*2-phenylpropanal by wild-type *Ct*XR and the substrate-binding-site mutants.

**Table 1:** Apparent kinetic parameters of wild-type *Ct*XR and substrate-binding-site mutants in the NADH-dependent reduction of racemic and (*S*)*-*2-phenylpropanal^a^

	*rac*-2-phenylpropanal		(*S*)-2-phenylpropanal		Optical preference ratio
*Ct*XR	*k*_cat_/*K*_m,*rac*_ (s^−1^ M^−1^)	*K*_m,*rac*_ (µM)	*k*_cat_/*K*_m,*S*_ (s^−1^ M^−1^)	*K*_m,*S*_ (µM)	(*S*)-aldehyde / racemic aldehyde
wild-type	130	350	160	450	1.23
D51A	28·10^3^	170	43·10^3^	120	1.54
W24F	13^b^	N/A	12	N/A	0.92
W24Y	10^b^	N/A	9	N/A	0.90
N310A	88	280	68	330	0.77
N310D	no activity	no activity	no activity	no activity	-

^a^The kinetic parameters were obtained using non-linear least-squares fitting of the experimental data to the Michaelis–Menten equation in SigmaPlot 2006 (version 10.0 for Windows). ^b^When limited substrate solubility prevented saturation of the enzyme, *k*_cat_/*K*_m_ was calculated from the slope of the Michaelis–Menten plot where the rate is linearly dependent on the substrate concentration. N/A not applicable

*Racemic substrate*. The wild-type enzyme showed a *K*_m,rac_ value of 350 µM and a *k*_cat_ value of 0.05 s^−1^, corresponding to a specificity constant (*k*_cat_/*K*_m,rac_) of 130 s^−1^ M^−1^. The mutants W24F and W24Y displayed only 8 to 10% of the wild-type activity. Although the N310A mutant had a specificity constant similar to the wild type, the N310D mutant showed no activity. *Ct*XR D51A stood out with a *K*_m,rac_ value of 170 µM and a *k*_cat_ value of 4.8 s^−1^, corresponding to a *k*_cat_/*K*_m,rac_ of 28·10^3^ s^−1^ M^−1^. Hence, replacement of the charged aspartic acid with alanine led to 215-fold higher catalytic efficiency compared to the wild type. Conversely, introduction of an additional aspartic acid in the substrate-binding pocket (N310D) completely abolished enzyme activity with 2-phenylpropanal. Enlargement of the substrate binding pocket by replacement of the bulky Trp-24 decreased the enzyme's activity towards 2-phenylpropanal*.*

*(S)-2-phenylpropanal*. Kinetic parameters obtained with the racemic substrate and (*S*)*-*2-phenylpropanal were used to calculate the optical preference ratio (*k*_cat_/*K*_m,S_)/(*k*_cat_/*K*_m_*,*_rac_) (Table 1). The wild type showed a ratio of 1.23 with a preference for the *S*-enantiomer. The D51A mutant showed a ratio of 1.54 and hence a stronger preference for the *S*-enantiomer. The W24F, W24Y and N310A mutants displayed ratios < 1, suggesting a preference for the *R*-enantiomer. It should be noted that the *Ct*XR mutants showed 35 to 100-fold reduced catalytic activity towards the natural substrate xylose [24].

We used isolated *Ct*XR D51A in bioreductions of 0.5 mM *rac*-2-phenylpropanal. The substrate (log*P* 2.11, https://scifinder.cas.org/) displayed a maximal solubility of 0.5 mM in the buffer (50 mM potassium phosphate, pH 7.0 with 25% DMSO). Product concentrations and *ee* values obtained at enzyme concentrations between 360 and 0.6 U/mL are listed in Table 2. The product *ee* values increased with decreasing amounts of the enzyme. Unexpectedly, the reactions stopped after approximately 1 h and maximal product concentrations of only ~ 80 µM were achieved (for a time course see the Additional file 1: Fig. S1). We suspected that enzyme deactivation caused the low conversions and decided to use a whole-cell biocatalyst expressing *Ct*XR D51A for further experiments.

**Table 2:** Conversions and product *ee*-values for the reduction of *rac*-2-phenylpropanal by isolated *Ct*XR D51A.^a,b^

*Ct*XR (U/mL)^c^	Phenylpropanol (µM)	*ee* (*S*)-Phenylpropanol (%)
D51A (20)	76 ± 3	41 ± 4
D51A (3.4)	51 ± 5	98 ± 2
D51A (0.6)	13 ± 5	99.1 ± 0.3

^a^NAD^+^ concentration 0.7 mM, reaction time 2 h. ^b^The phenylpropanol concentrations were measured by HPLC, the data represent the mean values of two reaction replicates and standard deviations from the mean. ^c^The enzyme activity (U/mL) was measured with 0.5 mM *rac*-2-phenylpropanal

### Optimization of *rac*-2-phenylpropanal reduction using whole cells of *E. coli* co-expressing *Ct*XR D51A and a yeast formate dehydrogenase

**Fig. 2:**
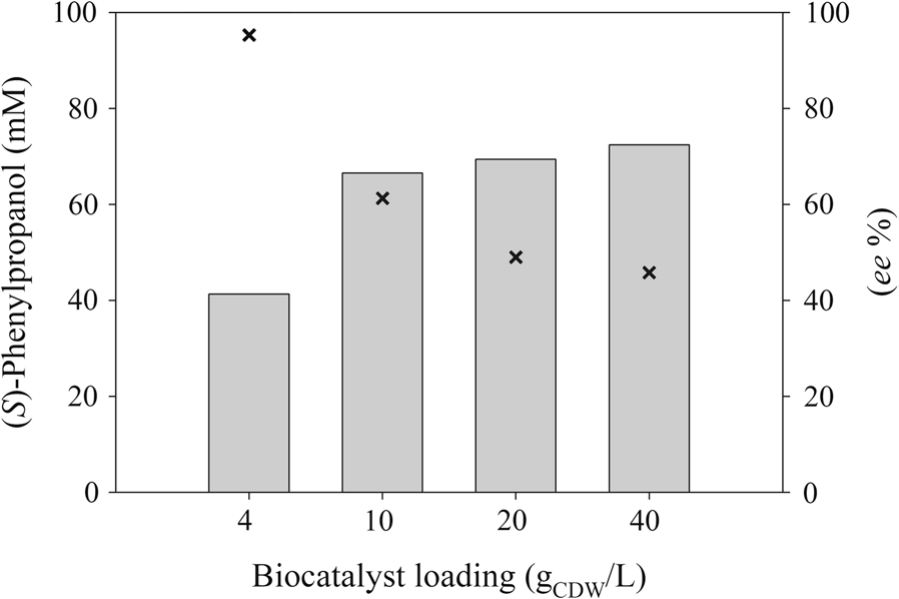
Conversions and product enantiopurities for the reduction of 100 mM racemic 2-phenylpropanal using a lyophilized and rehydrated whole-cell catalyst. The effects of catalyst loading on product concentration (mM, bars) and product *ee*-value (%, crosses) were studied. The NAD^+^ concentration was 6 mM and the reaction time was 24 h. (The details are summarized in the Supplementary data, Table S1)

Bioreductions were accomplished using whole cells of transgenic *E. coli* that were lyophilized and rehydrated in phosphate buffer. The phenylpropanal reductase and formate dehydrogenase activities of the whole-cell biocatalyst were 2200 and 154 U/g_CDW_, respectively (the *rac*-2-phenylpropanal reduction activity was determined with 0.5 mM *rac*-2-phenylpropanal). Addition of the biomass to reaction mixtures with a final substrate concentration of more than 100 mM led to emulsification [25]. A substrate concentration of 100 mM was converted by 4 and 10 g_CDW_/L of the whole-cell biocatalyst to 41 and 67 mM (*S*)-phenylpropanol with *ee*-values of 95.3 and 62%, respectively (Fig. 2). Next, we optimized the catalyst loading (20 and 40 g_CDW_/L), substrate concentration (1 and 2 M), NAD^+^ concentration (3, 6, 8, 10, 12 and 14 mM) and cyclodextrin addition (38 and 75 mM) (Table 3). Our primary goal was a high product concentration, as the substrate had shown a strong deactivating effect on the isolated enzyme. Secondary goals were high product enantiopurity and a high conversion ratio. We increased the substrate concentration to 1 M at 20 and 40 g_CDW_/L catalyst loading. Product enantiopurity and conversion increased with higher biocatalyst concentration. A catalyst loading of 20 g_CDW_/L led to an *ee* value of 95.1%, but the conversion ratio fell to 23%. Increasing the catalyst loading to 40 g_CDW_/L decreased the *ee* value to 92.2% but achieved 51% conversion (Table 3, entries with 3 mM NAD^+^). A substrate concentration of 2 M was too high, resulting in only low conversions of ~ 30%. The step-wise addition of substrate at 0, 2 and 4 h corresponding to a total substrate concentration of 1 M led to conversions and product *ee*-values comparable to batch conversions (Table 3). We added higher concentrations of the coenzyme NAD^+^ (6 to 14 mM) to reactions with 40 g_CDW_/L and 1 M substrate to further push the reaction towards full conversion. With 10 and 12 mM NAD^+^, conversion ratios of up to 84% were reached, with *ee* values of 92–93% (Table 3). The effects of catalyst loading and NAD^+^ concentration are summarized in the Additional file 1: Fig. S2*.* The addition of 75 mM 2-hydroxypropyl-β-cyclodextrin was previously shown to boost bioreductions [19]. Here, the addition of 38 or 75 mM 2-hydroxypropyl-β-cyclodextrin had a positive effect on reactions with a catalyst loading of 20 g_CDW_/L, but it had of no significant effect on bioreductions of 1 and 2 M 2-phenylpropanal (Table 3).

**Table 3:** Conversions and product ee-values for the reduction of rac-2-phenylpropanal using a lyophilized and rehydrated whole-cell biocatalyst. The effects of catalyst loading, substrate concentration, coenzyme concentration and the addition of HBC (2-hydroxypropyl-β-cyclodextrin) were investigated.^a,b^

Catalyst loading (g_CDW_/L)	*rac*-2-Phenylpropanal (M)	NAD^+^ (mM)	HBC (mM)	Phenylpropanol (mM)	*ee* of (*S*)-Phenylpropanol (%)
20	1	3	0	234 ± 16	95.1 ± 0.0
20	1	3	38	401 ± 10	94.1 ± 0.0
20	1	3	75	406 ± 2	93.3 ± 0.1
40	1	3	0	506 ± 14	92.2 ± 0.0
40	1	6	0	619 ± 24^c^	93.3 ± 1.1^c^
40	1 M fed-batch	6	0	611 ± 12	94.0 ± 0.1
40	1	8	0	662 ± 9	94.1 ± 0.1
40	1	10	0	843 ± 31	93.1 ± 0.2
40	1	12	0	839 ± 36	92.9 ± 0.1
40	1	14	0	765 ± 31	92.3 ± 0.1
40	1	6	38	634 ± 8	94.3 ± 0.2
40	1	6	75	598 ± 7	92.9 ± 0.2
40	2	6	0	571 ± 10	95.4 ± 0.1
40	2	6	38	633 ± 2	93.4 ± 0.5
40	2	6	75	637 ± 22	92.2 ± 0.2

^a^Reaction time 48 h. ^b^The data represent the mean values and deviations from the mean of two reaction replicates. ^c^The data represent the mean values and standard deviations of 7 reaction replicates

### Reproducibility, recovery and by-products

Replicate bioreductions (N = 7) with 40 g_CDW_/L and 6 mM NAD^+^ showed high reproducibility with a mean conversion ratio of 62% and a standard deviation of 2.4%. The enantiomeric excess was 93.3 ± 1.1%. The formation of broad peaks prevented the quantification of the aldehyde substrate by chiral reversed-phase HPLC. We therefore additionally analyzed the bioreduction samples by chiral GC-FID (Additional file 1: Fig. S4). The high reactivity of the substrate 2-phenylpropanal prompted us to investigate possible by-products generated through chemical or bio-chemical side-reactions. It was previously shown that acetophenone is formed through the degradation of *rac*-2-phenylpropanal by atmospheric oxygen [26]. Hydrophobic compounds were extracted from two reaction mixtures (1 M *rac*-phenylpropanal, 40 g_CDW_/L catalyst, 6 mM NAD^+^, 1 mL volume per reaction), after which the solvent, and possibly unreacted 2-phenylpropanal (bp 92 – 94°), were removed under reduced pressure. The concentrate with a weight of 203 mg was composed of 86% 2-phenylpropanol, 7% acetophenone and 7% ethyl acetate (extractant) according to ^1^H-NMR (Additional file 1: Fig. S5). We found trace amounts of 1-phenylethanol, the enzymatic reduction product of acetophenone [27]. Notably, no 2-phenylpropanal was found. The substrate was in a chemical equilibrium between *rac*-2-phenylpropanal and its corresponding hydrates. The previously reported enzymatic oxidation of 2-phenylpropanal hydrates to the corresponding carboxylic acids was not observed [12, 28], and no enol or aldol isoforms of the substrate were detected. The HPLC, GC and NMR analyses are shown in Additional file 1: Sects. 4, 5 and 6, respectively. The loss of substrate/product with the biomass was approximately 15% under the described reaction conditions (data not shown).

## Discussion

### Literature survey

A literature survey yielded 13 examples of enantioselective bioreductions of 2-phenylpropanal using free or immobilized enzymes (Table 4). Previous studies investigated bioreduction catalysts (soluble enzymes discussed in entries 1–5, 8–10, 12, 13; immobilized enzymes in entries 6,7,11) in the kinetic resolution of *rac*-2-phenylpropanal (entries 4, 6–11). Most enantioselective enzymes preferred the (*S*)-aldehyde (entries 1–8, 10). Rocha-Martín et al. [13] reported that the ADH from *Thermus thermophilus* HB27 exhibits anti-Prelog specificity (entry 11). Dong et al. [8] used directed evolution on an ADH from *Thermoanaerobacter brockii*, which displayed moderate Prelog-type selectivity, to improve the formation of (*S*)- and (*R*)-alcohols (entries 8, 9). HLADH was used in most studies due to its enantioselectivity, substrate tolerance up to a concentration of 165 mM, and usefulness in coupled substrate strategies (oxidation of cheap alcohols for NADH-recycling). Most other selective ADHs stem from thermophilic organisms and display intrinsically high stability in adverse reaction media. The low number of reported enzymes and the low obtained product titers indicated that the reactive aldehyde substrate generally inactivates the enzymes [29]. Remarkably, the often-used host *E. coli* shows native activity towards 2-phenylpropanal (entry 14) [30], which was traced back to the tetrameric ADH of *E. coli* (entry 12) [1, 31]. Buffered solutions containing water-soluble co-solvents (also used as sacrificial substrates for NADH recycling) were also used in many studies, whereby the aqueous phase enabled the racemization of the substrate. The highest published product concentration of 82 mM was achieved in a 47:63 buffer/isopropyl ether mixture (entry 4) [16].

**Table 4:** Literature survey of studies on the reductive enzymatic kinetic resolution of rac-2-phenylpropanal

Entry	Bioreduction catalyst, NAD(P)H-recycling strategy	*rac*-2-phenylpropanal	Reaction medium (auxiliary substrate)	Product (Conversion)	Enantiopurity	Aim of the study	Ref
	**Horse liver ADH (Zn-containing ADH)**						
1	Free enzyme, coupled substrate 1,4-butanediol	5 mM	Buffer pH 7.5, 1% v/v CH_3_CN, (2.5 mM 1,4-butanediol)	5 mM (98%)	95% *ee S*	Probing the enzyme's coenzyme recycling ability using the oxidation of 1,4-butanediol into the corresponding lactone	14
2	Free enzyme, coupled substrate ethanol	0.5 mM	Buffer pH 7.5, (0.5 M ethanol)	0.38 mM (75%)	98% *ee S*	Investigation of DKR, including substrate racemization velocity	10,15
3	Free enzyme, phenylpropanal oxidation for NADH-recycling	75 mM	Buffer pH 7.5; 4% v/v MTBE	28 mM (37%)	96% *ee S*	Investigation of a biocatalytic asymmetric disproportionation (biocatalytic Cannizzaro reaction)	12
4	Free enzyme, coupled substrate ethanol	165 mM	Buffer, 63% v/v isopropyl ether, (0.6 M ethanol)	82 mM (50%)	96% *ee S*	Probing the enzyme's enantioselectivity and coenzyme recycling ability	16
5	Free enzyme, coupled substrate ethanol	30 mM	Buffer pH 8, (5% v/v ethanol)	28 mM (93%)	93% *ee S*	One-pot, two-step reaction: oxidation of *rac*-2-phenyl-1-propanol into *rac*-2-phenylpropanal followed by a dynamic enantioselective bioreduction	1
6	Immobilized enzyme, coupled substrate ethanol	5 mM	Buffer pH 7.5, 50% v/v hexane, (0.5 M ethanol)	4.2 mM (84%)	> 98% *ee S*	Characterization of the immobilized catalyst	17
7	Immobilized enzyme, coupled substrate ethanol	300 mM	Isopropyl ether (saturated with buffer), 0.5% buffer pH 7.0, (1 M ethanol)	46 mM (15%)	95% *ee S*	Probing the enzyme's substrate scope and enantioselectivity in organic solvents	11
	**Thermostable ADHs (enzyme superfamily)**						
8	Free *Thermoanaerobacter brockii* LG296 ADH mutant (Zn-containing ADH), coupled substrate isopropanol	30 mM	Buffer pH 7.4, (20% v/v isopropanol)	23 mM (75%)	95% *ee S*	Development of enantioselective mutants	8
9	Free *Thermoanaerobacter brockii* LG277 ADH mutant (Zn-containing ADH), coupled substrate isopropanol	10 mM	Buffer pH 7.4, (20% v/v isopropanol)	7.5 mM (75%)	92% *ee R*	Development of enantioselective mutants	8
10	Free *Sulfolobus solfataricus* ADH-10 (Zn-containing ADH), coupled substrate ethanol	5 mM	Buffer pH 9, (5% ethanol)	3.7 mM (74%)	98% *ee S*	Probing the enzyme's substrate scope and enantioselectivity	9
11	Immobilized *Thermus thermophilus* ADH (short-chain dehydrogenases/reductase), coupled enzyme yeast formate dehydrogenase	1 mM	Buffer pH 7, 5% v/v CH_3_CN (0.1 M formic acid)	1 mM (100%)	71% *ee R*	Characterization of the immobilized catalyst	13
	**Other ADHs (enzyme superfamily)**						
12	Free *E. coli* ADH (Zn-containing ADH)	30 mM	Buffer pH 8, (5% v/v ethanol)	29 mM (97%)	94% *ee S*	One-pot, two-step reaction: oxidation of *rac*-2-phenyl-1-propanol into *rac*-2-phenylpropanal followed by a dynamic enantioselective bioreduction	1
	**Origin of enzyme not stated**						
13	Free Evo-1.1.200 from Evocatal coupled substrate ethanol	30 mM	Buffer pH 9, (5% v/v isopropanol)	29 mM (95%)	89% *ee R*	One-pot, two-step reaction: oxidation of *rac*-2-phenyl-1-propanol into *rac*-2-phenylpropanal followed by a dynamic enantioselective bioreduction	1
	**Whole-cell catalysts**						
14	*E. coli* JM109, NAD(P)H-recycling by native microbial metabolism	22 mM	M9 medium, 30% v/v organic phase (9:1 isopropyl ether: isooctane)	4.4 mM (20%)	~ 50% *ee* (*?* unknown)	Probing the host background activity	30
15	*Ct*XR D51A mutant (aldo–keto reductase) coupled enzyme yeast formate dehydrogenase	1000 mM	Buffer pH 7.5, (1.05 M formic acid)	843 mM (98.8%, but product and substrate loss)	93.1% *ee S*	Process optimization for imroved enantiopurity and yield	This work

### Reduction of *rac*-2-phenylpropanal by *Ct*XR variants

We investigated five enzyme variants with single point mutations in close vicinity to the stereocenter of 2-phenylpropanal (Table 1). Replacement of the charged Asp-51 with alanine substantially improved the enzyme's activity. The catalytic efficiency increased 215-fold for *rac*-2-phenylpropanal and 270-fold for the (*S*)-aldehyde compared to the wild type (Table 1). Similarly, the catalytic efficiency in the reduction of *o*-chloroacetophenone was improved 13-times by the D51A mutation [19], which was accompanied by a 50-fold decrease in the conversion of the native substrate xylose. Replacement of aspartate with alanine appears to be a general strategy for improving the activity of *Ct*XR and its homologues towards hydrophobic substrates [24]. By contrast, the W24F and W24Y mutations diminished the reductase activity in the conversion of *rac*-2-phenylpropanal to 6 and 8% of the value of wild-type *Ct*XR, respectively (Table 1). Reduced enzyme activities (with higher *K*_m_-values) towards *rac*-2-phenylpropanal were observed following the removal of the bulky Trp-24, which confirmed a general role of Trp-24 in the efficient conversion of aldehydes. The effects of these single point mutations on the enantioselectivity are discussed below.

### Enantioselectivity of *Ct*XR variants towards 2-phenylpropanal

**Fig. 3:**
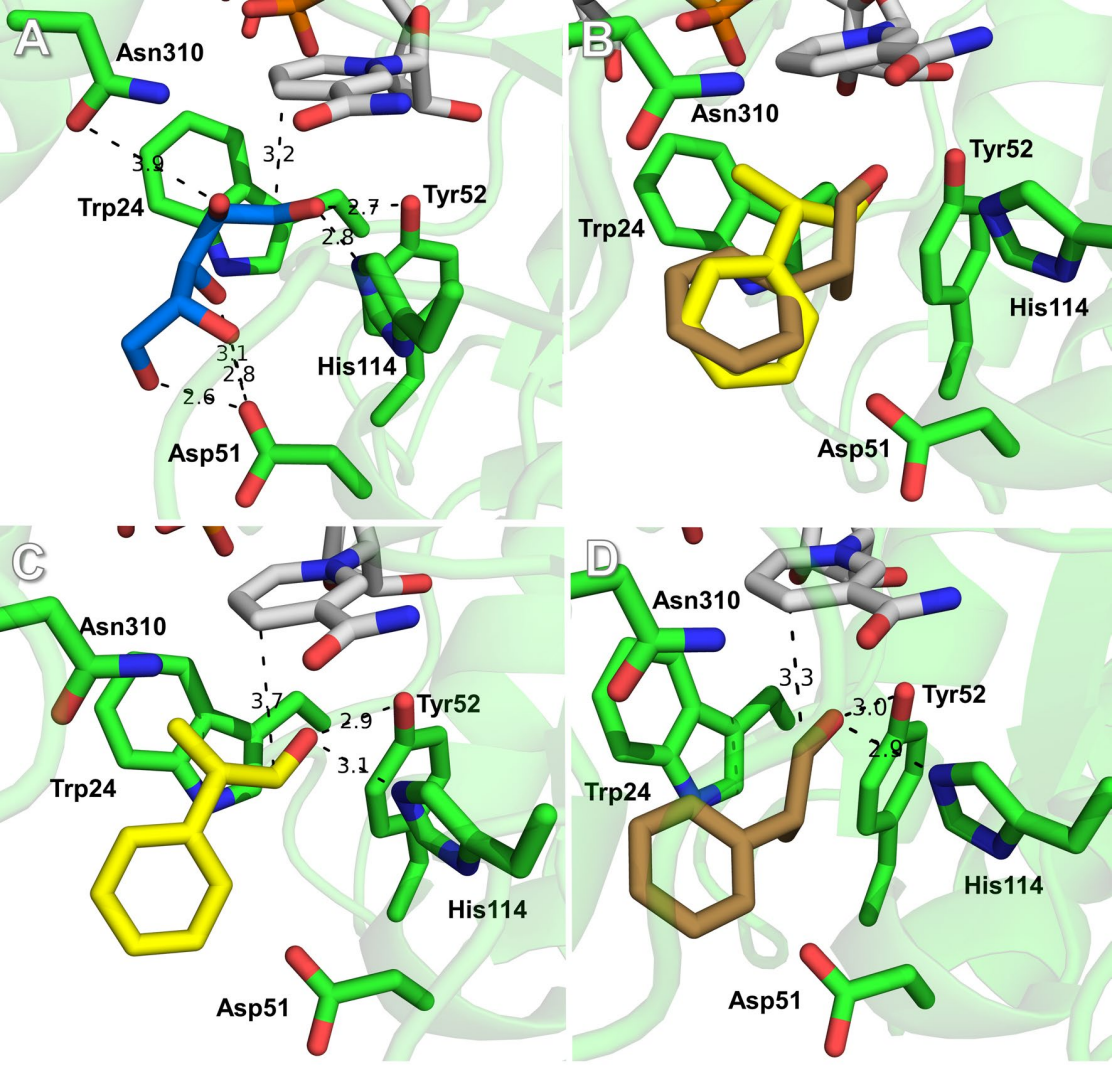
Active site of wild-type CtXR with NAD^+^ (PDB 1MI3, [33]) and modelled substrates. **A** Xylose (blue carbons, red oxygens), **B** (*S*)- and (*R*)-2-phenylpropanal (*S*-enantiomer yellow carbons, *R*-enantiomer brown carbons, red oxygens), **C** (*S*)-2-phenylpropanal (yellow carbons, red oxygen), **D** (*R*)-2-phenylpropanal (brown carbons, red oxygen). Possible hydrogen bonds between substrates and the enzyme are shown as dashed lines, with distances in Å

The enantioselectivity of an enzyme is generally defined by the ratio of catalytic efficiencies for the two enantiomers (E = (*k*_cat_/*K*_m,S_)/(*k*_cat_/*K*_m_*,*_R_) [32]. The published racemization velocity (*k*_rac_) of 2-phenylpropanal is 75·10^–6^ s^−1^, corresponding to a half-life (t_1/2_) of ~ 2 h [15]. The relatively slow racemization should generally enable the determination of the *k*_cat_/*K*_m,enantiomer_ values using enzymatic assays that can be completed in 5 min. However, we observed slow racemization of the pure enantiomers during frozen storage at -18 °C (data not shown). Hence, the enantioselectivities shown in Table 1 (expressed as the (*k*_cat_/*K*_m,S_)/(*k*_cat_/*K*_m,rac_) ratios) represent approximate values that are still useful to guide enzyme selection and reaction optimization. The wild type showed a preference for the (*S*)-aldehyde (ratio of 1.23). The D51A mutant showed a markedly stronger preference for the *S*-enantiomer than the N310A mutant (1.54 vs. 0.77). Asp-51 and Asn-310 are on opposite sides of the substrate-binding pocket (Fig. 3). Asp-51 is suggested to interact with the hydroxyl groups of the natural substrate xylose at C-3, C-4 and C-5, while Asn-310 interacts with the hydroxyl group at C-2 [23] (Fig. 3A). Docking simulations of wild-type *Ct*XR (in complex with NAD^+^) with (*S*)- and (*R*)-2-phenylpropanal are shown in Fig. 3B. Differential positions of the (*S*)- and (*R*)-2-phenylpropanal enantiomers provide a possible explanation for the enantiomer preferences of the mutants. Replacement of Asp-51 with alanine might introduce an interaction between the alanine and the phenyl-ring of (*S*)-2-phenylpropanal (Fig. 3C). After replacement of Asn-310 with alanine, this interaction between alanine and the phenyl-ring of (*R*)-2-phenylpropanal becomes even more plausible (Fig. 3D). The W24F and W24Y mutants showed (*k*_cat_/*K*_m,S_)/(*k*_cat_/*K*_m_*,*_rac_) ratios of ~ 0.91 (Table 1). along with an approximately ≥ tenfold reduction of their catalytic efficiency. Lower activities (with higher *K*_m_-values) and a preference for the (*R*)-aldehyde might indicate an even poorer interaction between the aromatic rings of phenylalanine or tyrosine and the aldehyde proton of the (*S*)-substrate. In conclusion, the D51A mutant of *Ct*XR was identified as a variant with improved catalytic efficiency and enantioselectivity, exhibiting a ~ 40-fold higher *k*_cat_ and a ~ 30-fold smaller *K*_m_ for the racemic substrate compared to published values for variants of the ADH from *Thermoanaerobacter brockii* [8].

### Catalyst stabilization

*Ct*XR is generally known to have low substrate tolerance, with half-lives in the presence of 5 to 10 mM *o*-chloroacetophenone or 1-(2-chlorophenyl)ethanol shorter than 3 min. In the case of *o*-chloroacetophenone and its reaction product, the unfavorable logP values (~ 2) are at least partially responsible for the fast enzyme deactivation. Integration of this oxidoreductase into whole-cell biocatalysts (*E. coli*, *S. cerevisiae*, *C. tenuis*) was previously shown to substantially stabilize the enzyme, resulting in > tenfold improvements of the product titer [34]. Reactive aldehydes are known to form adducts with lysine, histidine and cysteine residues of proteins [29]. Fast enzyme deactivation by 2-phenylpropanal and *o*-chloroacetophenone might therefore be caused by different mechanisms. The use of whole cells provided an extreme case of catalyst stabilization in the presence of 2-phenylpropanal, since the isolated enzyme was deactivated by 0.5 mM aldehyde whereas the whole-cell catalyst was able to tolerate and convert 1 M substrate. Stabilization of the enzyme by whole cells and cell debris was previously reported for the synthesis of (*R*)‐phenylacetyl carbinol from benzaldehyde and pyruvate using a pyruvate decarboxylase from *Candida utilis*. The stabilization was ascribed to membrane components that form a microenvironment around the enzyme and thereby decrease aldehyde transfer to the enzyme, protecting it from deactivation at the aqueous/organic interphase [35]. The addition of cyclodextrin (HBC), which was previously shown to boost the reduction of *o*-chloroacetophenone, only had a minor effect on the lyophilized *E. coli* cells used in this study. Catalyst stabilization by cell components was apparently dominant, especially at the higher catalyst loading of 40 g_CDW_/L, and the addition of HBC only increased the product concentration at a lower catalyst loading of 20 g_CDW_/L (Table 3).

### Product enantiopurity and concentration

**Fig. 4:**
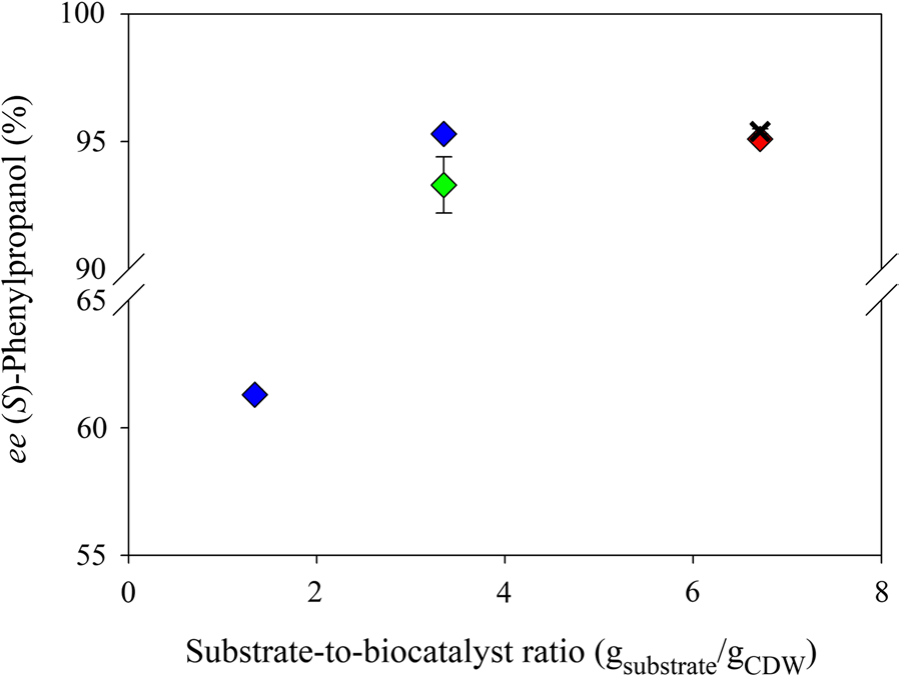
Effect of the substrate-to-biocatalyst ratio (whole-cell biocatalyst) on product enantiopurity. Blue diamonds show the conversion of 100 mM *rac*-2-phenylpropanal with 4 and 10 g_CDW_ biocatalyst, green diamonds with 1 M substrate and 40 g_CDW_, red diamonds with 1 M substrate and 20 g_CDW_, black crosses with 2 M substrate and 40 g_CDW_. (Data with error bars from reactions with 6 mM NAD^+^ are depicted. See also the section Optimization of 2-phenylpropanal bioreduction, Table 3)

Generally, an enzyme with higher enantioselectivity will generate a product with higher enantiomeric excess. In the case of an enzyme that converts one enantiomer much faster than the other, the product of the initial stage of the conversion at a high substrate and low product concentration will show high enantiopurity, whereas a decrease is expected during the course of the reaction (expressed in the Chen equation, [32]). Hence, product enantiopurity depends on the enantioselectivity of the reductase and on the availability (concentrations) of both substrate enantiomers. DKR strategies make use of the relatively fast racemization of 2-aryl-1-propanals (reviewed in Kourist et al. [4]). Optimally, there is an equal availability of both substrate enantiomers during the entire DKR reaction (both substrate enantiomers are present in equal concentrations). However, in the present case, a relatively high reductase activity was used to compensate for fast catalyst deactivation, leading to a trade-off between the enantiomeric purity and final titer of the product (Fig. 2). A dependence of product enantiopurity on reaction progress, especially at high enzyme loading, was also reported in other studies on DKR. The substrate racemization velocity was previously identified as the main factor limiting product enantiopurity [12]. Here, the substrate-to-catalyst ratio was identified as the main factor determining product enantiopurity. The *ee* value of the product showed the expected dependence on the substrate-to-catalyst ratio (expressed as g_substrate_/g_CDW_ in Fig. 4). The highest *ee*-values of 95.3 and 95.4 were obtained for the conversions of 100 and 2 M *rac*-2-phenylpropanal by 4 and 40 g_CDW_/L, respectively. However, the reduction of 2 M substrate was compromised by a low conversion ratio (~ 30%, Table 3). Thus, a compromise between product enantiopurity and the conversion ratio had to be found. Overall, the main limiting factors were substrate racemization velocity, catalyst stability and catalyst loading, with higher catalyst loading leading to a loss of the hydrophobic product in the biomass fraction during downstream processing [36].

## Conclusions

Detailed optimization of a reductive enzymatic dynamic kinetic resolution of racemic 2-phenylpropanal yielded 843 mM (115 g/L) (*S*)-2-phenylpropanol with 93% *ee*. The multilevel strategy included rational mutagenesis of *Ct*XR for improved enzyme activity and enantioselectivity, the use of an *E. coli* whole-cell catalyst for enzyme stabilization and coenzyme recycling, as well as the optimization of the substrate-to-catalyst ratio to increase the enantiopurity and final titer of the product. The use of the whole-cell catalyst led to a remarkable > 1000-fold improvement of the product concentration, indicating strong enzyme stabilization by cellular components. The most important factor for obtaining high enantiopurities and product concentrations was the ratio of substrate to catalyst (Fig. 4). High reduction velocities led to high conversions at the expense of lower product enantiopurities. The conversion with a catalyst loading of 40 g_CDW_, 10 mM NAD^+^ and 1 M substrate was identified as a suitable compromise, affording 843 mM (*S*)-phenylpropanol with 93.1% *ee* (Table 3).

## Materials and methods

### Chemicals, enzymes and strains

Racemic 2-phenylpropanal (98%), racemic 2-phenylpropanol (97%), acetophenone (99%) and racemic 1-phenylethanol (≥ 98%) were purchased from Sigma-Aldrich/Fluka (Vienna, Austria); (*S*)-2-phenylpropanal (95%) and (*R*)-2-phenylpropanal (95%) were from Accela (Prien – Chiemsee, Germany); 2-hydroxypropyl-β-cyclodextrin (HBC, batch number OH053931501) was from Carbosynth (Berkshire, UK); NAD^+^ (98%), acetonitrile (≥ 99) and ethyl acetate (≥ 99,9%) were from Roth (Karlsruhe, Germany). Other chemicals were from Sigma-Aldrich/Fluka or Roth, and were of the highest purity available. Materials for genetic modification were reported elsewhere [24]. The used reductases were the wild type and single-point mutants of *Candida tenuis* xylose reductase (*Ct*XR wild-type GenBank ID AF074484). Site-directed mutagenesis for the construction of the *Ct*XR mutants D51A, W24F, W24Y, N310A, and N310D was carried out using inverse PCR as described elsewhere [24]. The protein expression of *Ct*XR (wild-type and mutants D51A, W24F, W24Y, N310A, N310D) was described previously [24]. An *E. coli* Rosetta2 strain co-expressing *Ct*XR D51A and *Cb*FDH (GenBank ID AJ011046) was used in the bioreductions. The construction of the co-expression strain was previously described by Rapp et al. [19]. Cultivation of the co-expression strain was described previously [19], and is summarized in the Additional file 1. The biomass was frozen at −20 °C, lyophilized (Christ α 1–4 lyophilizer from Braun Biotech International) and stored at −20 °C.

### Enzyme kinetics

Steady state kinetic parameters for the NADH-dependent reduction of 2-phenylpropanal by *Ct*XR variants were determined spectrophotometrically as described earlier [24]. The solubility of 2-phenylpropanal in water was increased to 0.5 mM by the addition of 25% v/v DMSO. Substrate solutions were freshly prepared and immediately used to avoid non-enzymatic decomposition or racemization in aqueous solution in the case of (*S*)-2-phenylpropanal. A typical measurement period was 5 min. Non-specific background activity was taken into account by measuring blank mixtures. The added DMSO had no effect on the enzyme's activity with the natural substrate D-xylose.

### Bioreduction of racemic 2-phenylpropanal

#### Reduction by the isolated CtXR D51A

Racemic 2-phenylpropanal was dissolved in DMSO prior to dilution into 50 mM potassium phosphate buffer, pH 7.0, to a final DMSO concentration of 25%. The substrate (final concentration 0.5 mM) was incubated at 25 °C in the presence of 0.2 mM NADH and *Ct*XR D51A for 2 h. For time-course analysis, 100-µL samples were taken from the reaction mixtures (final reaction volume 1.5 mL) at the specified timepoints. All samples were diluted 1:1 with acetonitrile and centrifuged prior to analysis by chiral HPLC.

#### Whole-cell bioreductions

Lyophilized biomass (40 mg or 80 mg) was rehydrated in 1 mL of potassium phosphate buffer (100 mM, pH 6.2) containing NAD^+^ (0.5−14 mM) and sodium formate (50 mM, excess over substrate) in 2 mL Eppendorf tubes. Activities of the whole biomass, measured after cell lysis and protein extraction for *rac*-2-phenylpropanal reduction and formate dehydrogenase were 2200 and 154 U/g_CDW_, respectively [19]. *rac*-2-phenylpropanal reduction activity was determined at 0.5 mM *rac*-2-phenylpropanal in 50 mM potassium phosphate buffer, pH 7.0, with 25% DMSO. The rehydrated biomass (containing NAD^+^ and sodium formate, volume of the biomass slurry ≤ 50% v/v of the total bioreduction mixture) was combined with the substrate and filled up to a total working volume of 1 mL. In case of cyclodextrin-aided conversions, HBC and substrate were weighed separately in Eppendorf tubes containing 50 µL buffer, followed by vortexing prior to addition to the biomass. Eppendorf tubes were sealed with parafilm and vortexed until the mixtures completely emulsified. The mixtures were reacted for 24 or 48 h at room temperature using an end-over-end rotator (30 rpm). The fed-batch bioreductions were started as batch reactions with 330 mM substrate, 6 mM NAD^+^ and 40 g_CDW_/L. 50 µL *rac*-2-phenylpropanal, and were fed after 2 and 4 h to reach a total substrate concentration corresponding to 1 M. The reaction was carried out for 48 h.

#### Substrate/product recovery

For recovery experiments at substrate/product concentrations of 1 M, reaction mixtures containing 20 or 40 g_CDW_/L catalyst were prepared without adding NAD^+^. The samples were incubated under the same conditions as used for biotransformations and extracted following the procedure described in Sect. Analytical methods. Substrate/product recovery was performed in duplicates.

### Analytical methods

For HPLC and GC analyses, ethyl acetate (1 mL) was added to 1 mL of a reaction mixture in a 2 mL Eppendorf tube. The tubes were vortexed and the mixtures transferred into 15 mL Sarstedt tubes. The tubes were then filled up to 10 mL with ethyl acetate, vortexed and centrifuged for 15 min, 25 °C and 3220 g for extraction. Final dilutions in ethyl acetate contained 5 mM substrate/product. For NMR analyses, substrate/product present in ethyl acetate after extraction was transferred into round-flasks and evaporated under reduced pressure. The isolated substrate/product was directly dissolved in deuterated methanol (20 µL substrate/product + 680 µL solvent).

#### Chiral HPLC analysis

HPLC analysis was performed using a Merck-Hitachi LaChrom HPLC system equipped with a Merck L-7490 RI detector, an L-7400 UV-detector, a reversed phase Chiralpak® AD-RH column (from Daicel, obtained at Sigma Aldrich, Vienna, Austria) and a thermostat column oven (40 °C). The mobile phase was composed of 25% acetonitrile in ddH_2_O at a flowrate of 30 mL/min. HPLC standard curves were prepared using racemic product at 0.1, 0.5, 1, 5, and 10 mM concentrations. Peak areas at corresponding retention times were used to calculate the concentrations. The enantiomeric excess of the major product (*S*)-2-phenylpropanol was calculated using the formula *ee* = (*S*-alcohol – *R*-alcohol)/(*S*-alcohol + *R*-alcohol). The retention times and chromatograms of authentic standards (main products ((*S*)-2-phenylpropanol, (*R*)-2-phenylpropanol), by-products (acetophenone, phenylethanol) were summarized in the Additional file 1: Sect. 4. The aldehydes gave too broad peaks on HPLC and were quantified by GC-FID.

#### Chiral GC-FID analysis

GC analysis was performed on an Agilent 7890A GC with FID detection [12] equipped with a chiral Hydrodex®-β-TBDAc column with 25 m length and an inner diameter of 0.25 mm (from Macherey–Nagel obtained from FisherScientific, Austria, Vienna). The carrier gas was H_2_ with a flow of 1 mL/mi, the injection volume was 5 µL, the split ratio was 50:1, the inlet temperature 230 °C, and the detector temperature 250 °C. The following temperature program was used for the separation of analytes: 110 °C/hold 10 min; 2 °C per min to 123 °C/hold 3 min; 10 °C per min to 200 °C/hold 1 min. The retention times and chromatograms of authentic standards (main products ((*S*)-2-phenylpropanol, (*R*)-2-phenylpropanol), substrate ((*S*)-2-phenylpropanal, (*R*)-2-phenylpropanal) by-products (acetophenone, phenylethanol) are summarized in the Additional file 1: Sect. 5.

#### NMR analysis

^1^H-NMR spectra of isolated substrate/product from biotransformations (78% conversion) were recorded using a 300 MHz Bruker NMR unit (300 MHz for ^1^H) at 300 K. Chemical shifts (δ) were depicted in ppm relative to the resonance of the solvent (MeOD) (see also Additional file 1: Sect. 6).

### Enzyme–substrate docking simulations

PyMOL Molecular Graphics System (Open-Source, Schrödinger, LLC) was used for enzyme/ligand structure depictions. Ligand docking simulations were performed in YASARA (YASARA Biosciences GmbH, Vienna, Austria) using AutoDock Vina [37] with standard parameters.

## Supplementary Information

**Additional file 1.** 1. Biomass production. 2. Reduction of *rac*-2-phenylpropanal by isolated *Ct*XR D51A. Time course of the reduction of 0.5 mM substrate with 240 U/mL of isolated *Ct*XR D51A. 3. Biotransformation of *rac*-2-phenylpropanal. Conversions and product *ee*-values from the reduction of 100 mM *rac*-2-phenylpropanal using a lyophilized and rehydrated whole-cell catalyst. Effects of catalyst loading. Conversions and product *ee*-values from the reduction of 1 M *rac*-2-phenyl-propionaldehyde using a lyophilized and rehydrated whole-cell catalyst. Effects of catalyst loading (20 g_CDW_/L, 40 g_CDW_/L) and coenzyme concentration (NAD^+^ 3–14 mM). (The data are based on HPLC measurements, Table 3). 4. Reversed phase chiral HPLC. Separation of main products and by-products by HPLC. UV traces and retention times for *rac*-2-phenylpropanal, acetophenone, 1-phenylethanol, (*R,S*)-2-phenylpropanol, reaction buffer with NAD^+^, reaction buffer, bioreduction sample of 1 M *rac*-2-phenylpropanal reacted with 40 g_CDW_/L and 6 mM NAD^+^. 5. Chiral GC-FID. GC traces and retention times for main products, by-products and bioreduction replicates (N = 6) of 1 M *rac*-2-phenylpropanal reacted with 40 g_CDW_/L and 6 mM NAD^+^. 6. ^1^H-NMR. ^1^H-spectrum of the isolated product from a reaction with 78% analytical yield (HPLC) from 1 M *rac*-2-phenylpropanal reacted with 40 g_CDW_/L and 6 mM NAD^+^.

## Data Availability

The datasets used and/or analyzed during the current study are available in the Supplementary data or from the corresponding author upon reasonable request.

## Abbreviations

ADH: Alcohol dehydrogenase
*Cb*FDH: *Candida boidinii* Formate dehydrogenase
*Ct*XR: *Candida tenuis* Xylose reductase
DKR: Dynamic kinetic resolution
g_CDW_: Cell dry weight in gram
HBC: 2-Hydroxypropyl-β-cyclodextrin
HLADH: Horse liver alcohol dehydrogenase
*rac*: Racemic
