# Supplementary material for: Reductive enzymatic dynamic kinetic resolution affording 115 g/L (S)-2-phenylpropanol
Source: BMC Biotechnol. 2021 Oct 11;21:58. doi: 10.1186/s12896-021-00715-5 (PMC8507385; doi:10.1186/s12896-021-00715-5)
Supplement: Supplementary file 1 — Additional file 1. 1. Biomass production. 2. Reduction of rac-2-phenylpropanal by isolated CtXR D51A. Time course of the reduction of 0.5 mM substrate with 240 U/mL of isolated CtXR D51A. 3. Biotransformation of rac-2-phenylpropanal. Conversions and product ee-values from the reduction of 100 mM rac-2-phenylpropanal using a lyophilized and rehydrated whole-cell catalyst. Effects of catalyst loading. Conversions and product ee-values from the reduction of 1 M rac-2-phenyl-propionaldehyde using a lyophilized and rehydrated whole-cell catalyst. Effects of catalyst loading (20 gCDW/L, 40 gCDW/L) and coenzyme concentration (NAD+ 3–14 mM). (The data are based on HPLC measurements, Table 3). 4. Reversed phase chiral HPLC. Separation of main products and by-products by HPLC. UV traces and retention times for rac-2-phenylpropanal, acetophenone, 1-phenylethanol, (R,S)-2-phenylpropanol, reaction buffer with NAD+, reaction buffer, bioreduction sample of 1 M rac-2-phenylpropanal reacted with 40 gCDW/L and 6 mM NAD+. 5. Chiral GC-FID. GC traces and retention times for main products, by-products and bioreduction replicates (N = 6) of 1 M rac-2-phenylpropanal reacted with 40 gCDW/L and 6 mM NAD+. 6. 1H-NMR. 1H-spectrum of the isolated product from a reaction with 78% analytical yield (HPLC) from 1 M rac-2-phenylpropanal reacted with 40 gCDW/L and 6 mM NAD+. [file 12896_2021_715_MOESM1_ESM.docx]

**Supplementary data**

**Reductive enzymatic dynamic kinetic resolution affording 115 g/L *(S)*-2-phenylpropanol**

Christian Rapp^a^, Simone Pival^a,c^, Erika Tassano^b^, Bernd Nidetzky^a,c^, Regina Kratzer^a^*

^a^Institute of Biotechnology and Biochemical Engineering, Graz University of Technology, NAWI Graz, 8010 Graz, Austria. ^b^Department of Chemistry, University of Graz, Heinrichstrasse 28, NAWI Graz, 8010 Graz, Austria. ^c^Austrian Centre of Industrial Biotechnology (acib), 8010 Graz, Austria.

*Corresponding author phone +43 316 873 8412, e-mail: regina.kratzer@tugraz.at

**Content**

**1. Biomass production**

**2. Reduction of *rac*-2-phenylpropanal by isolated D51A *Ct*XR**

*Figure S1*. Time course of the reduction of 0.5mM substrate with 240U/mL of isolated D51A *Ct*XR. Blue dots (*S*)-2-phenylpropanal, orange dots (*R*)-2-phenylpropanol.

**3. Biotransformation of *rac*-2-phenylpropanal**

*Table S1 (data for Figure 2).* Conversions and product *ee*-values for the reduction of 100mM *rac*-phenylpropanal using a lyophilized and rehydrated whole-cell catalyst. Effects of catalyst loading (4, 10, 20, 40g_CDW_/L).

*Figure S2 (data in Table 3).* Conversions and product *ee*-values for the reduction of 1M *rac*-2-phenyl-propionaldehyde using a lyophilized and rehydrated whole-cell catalyst. Effects of catalyst loading (20, 40g_CDW_/L) and coenzyme concentration (NAD^+^ 3-14mM). The reaction time was 48 h. The data are based on HPLC measurements.

**4. Reversed phase, chiral HPLC**

*Figure S3*. Overlay of HPLC traces for *rac*-2-phenylpropanal (1), acetophenone (2), 1-phenylethanol (3), (*R,S*)-2-phenylpropanol (4), reaction buffer with NAD^+^ (5), reaction buffer (6), bioreduction sample from 1M 2-phenylpropanal reacted with 40g_CDW_/L and 6mM NAD^+^ (7).

*Table S2*. Retention times for main products and by-products obtained from reversed phase chiral HPLC.

**5. Chiral GC-FID**

*Figure S4*. Selected GC traces for bioreduction replicates (N=6) of 1M 2-phenylpropanal reacted with 40g_CDW_/L and 6mM NAD^+^.

*Table S3 (data corresponding to Figure S4)*. Retention times for main products and by-products obtained from chiral GC.

**6. ^1^H-NMR**

*Figure S5*. ^1^H-spectra in MeOD. The graph shows the spectrum of the isolated product of a reaction with 78 % analytical yield (HPLC). Spectra were recorded immediately after work-up. Signals ascribed to ethyl acetate are found at 1.75, and 3.9ppm, the signal ascribed to acetophenone is found at 2.45ppm.

**1. Biomass production**

An *E. coli* Rosetta2 strain co-expressing *Ct*XR D51A and *Cb*FDH (GenBank ID AJ011046) was used in the bioreductions. The cultivation and protein expression were done as follows.

Chloramphenicol and kanamycin were purchased from Sigma-Aldrich (Vienna, Austria); Ampicillin was obtained from Roth (Karlsruhe, Germany); all other chemicals were from Sigma-Aldrich/Fluka or Roth, and were of the highest purity available. The bacteria were grown in 1000 mL baffled shake flasks containing 200mL of LB medium supplemented with ampicillin (115mg/L), kanamycin (50mg/L) and chloramphenicol (34mg/L). The cultures were shaken at 130rpm and 37°C in a Certomat® BS-1 incubator (Sartorius) and cooled to 25°C after reaching an optical density of 1.1 (± 10%). Protein production was induced by adding isopropyl-β-D-thiogalactopyranoside (IPTG) to a final concentration of 0,25 mM and further 115mg/L of ampicillin. After 20h of cultivation, cells were harvested by centrifugation (Sorvall RC-5B). The biomass was frozen at -20°C, lyophilized (Christ α 1-4 lyophilizer from Braun Biotech International), and stored at -20°C.

**2. Time-course of the reduction of *rac*-2-phenylpropanal by isolated D51A *Ct*XR**

*Figure S1*. Time course of the reduction of 0.5mM substrate with 240U/mL of isolated D51A *Ct*XR (0.7mM NAD^+^). Blue dots (*S*)-2-phenylpropanal, orange dots (*R*)-2-phenylpropanol.

**3. Biotransformation of *rac*-2-phenylpropanal**

*Table S1 (data for Figure 2)*. Conversions and product *ee*-values for the reduction of 100mM *rac*-2-phenylpropanal using a lyophilized and rehydrated whole-cell catalyst. Effects of catalyst form and loading^a^.

| Whole-cell catalyst  (g_CDW_/L) | Conversion  (%) | *ee*  (%) | Ratio  (g_substrate_/g_CDW_) |
| --- | --- | --- | --- |
| 4 | 41.3 | 95.3 | 3.4 |
| 10 | 66.5 | 61.3 | 1.4 |
| 20 | 69.4 | 49.0 | 0.7 |
| 40 | 72.4 | 45.8 | 0.3 |

^a^NAD^+^ concentration 6mM, reaction time 24h. Data are based on HPLC measurements.

*
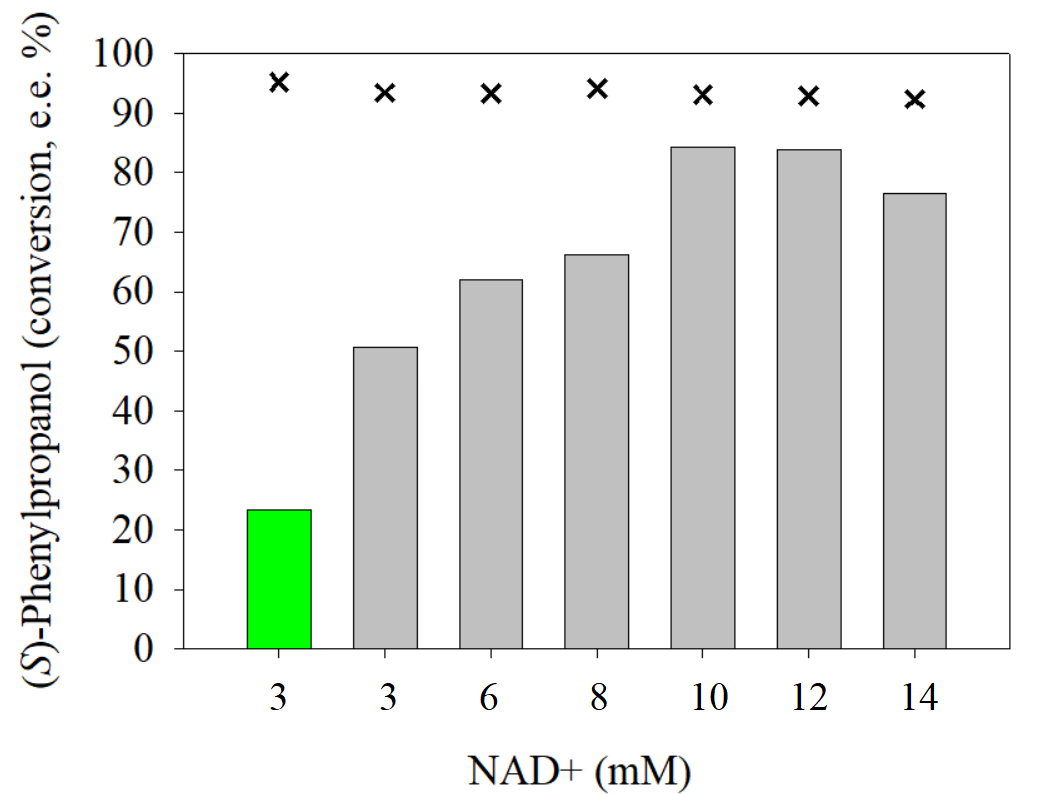
*

*Figure S2 (data in Table 3).* Conversions and product *ee*-values for the reduction of 1M *rac*-2-phenyl-propionaldehyde using a lyophilized and rehydrated whole-cell catalyst. Effects of catalyst loading (green bar 20g_CDW_/L, grey bars 40g_CDW_/L), coenzyme concentration (NAD^+^ 3-14 mM). The reaction time was 48 h. Data are based on HPLC measurements.

**4. Reversed phase chiral HPLC**

Separation of main products and by-products by HPLC (Chiralpak® AD-RH column from Daicel, mobile phase 25% acetonitrile in ddH_2_O, 40°C).


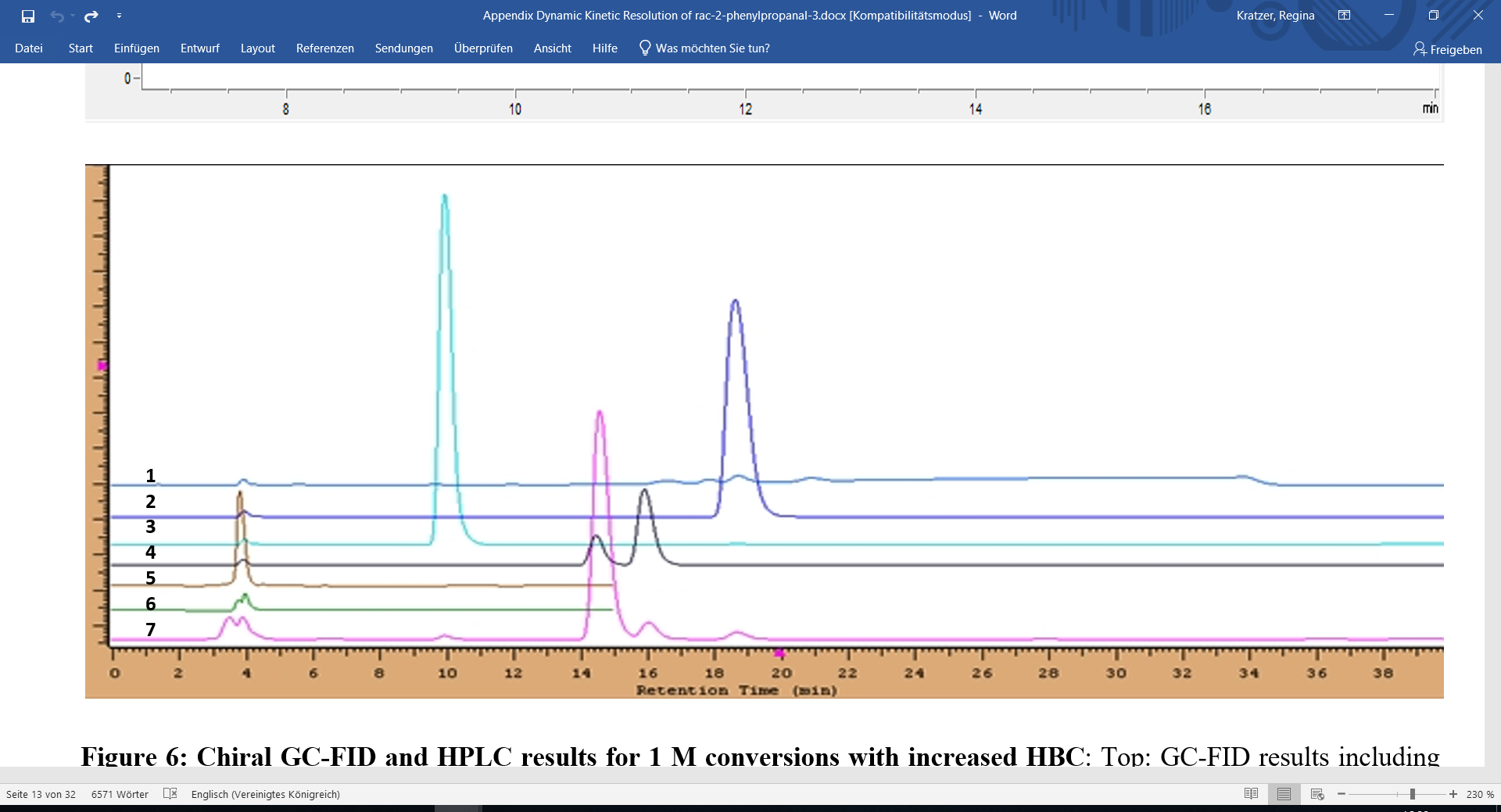


*Figure S3*. Overlay of HPLC traces for *rac*-2-phenylpropanal (1), acetophenone (2), *rac*-1-phenylethanol (3), (*R,S*)-2-phenylpropanol (4), reaction buffer with NAD^+^ (5), reaction buffer (6), bioreduction sample from 1M 2-phenylpropanal reacted with 40g_CDW_/L and 6mM NAD^+^ (7).

*Table S2 (data corresponding to Figure S3)*. Retention times of main products and by-products on reversed-phase chiral HPLC.

| Analyte | Retention time (min) |
| --- | --- |
| *rac*-2-Phenylpropanal | broad peak, not applicable |
| (*R*)-2-Phenylpropanol | 15.8 |
| (*S*)-2-Phenylpropanol | 14.5 |
| Acetophenone | 18.6 |
| *rac*-1-Phenylethanol | 9.9 (no separation) |

**5. Chiral GC-FID**

*(S)-*2-phenylpropanol

| **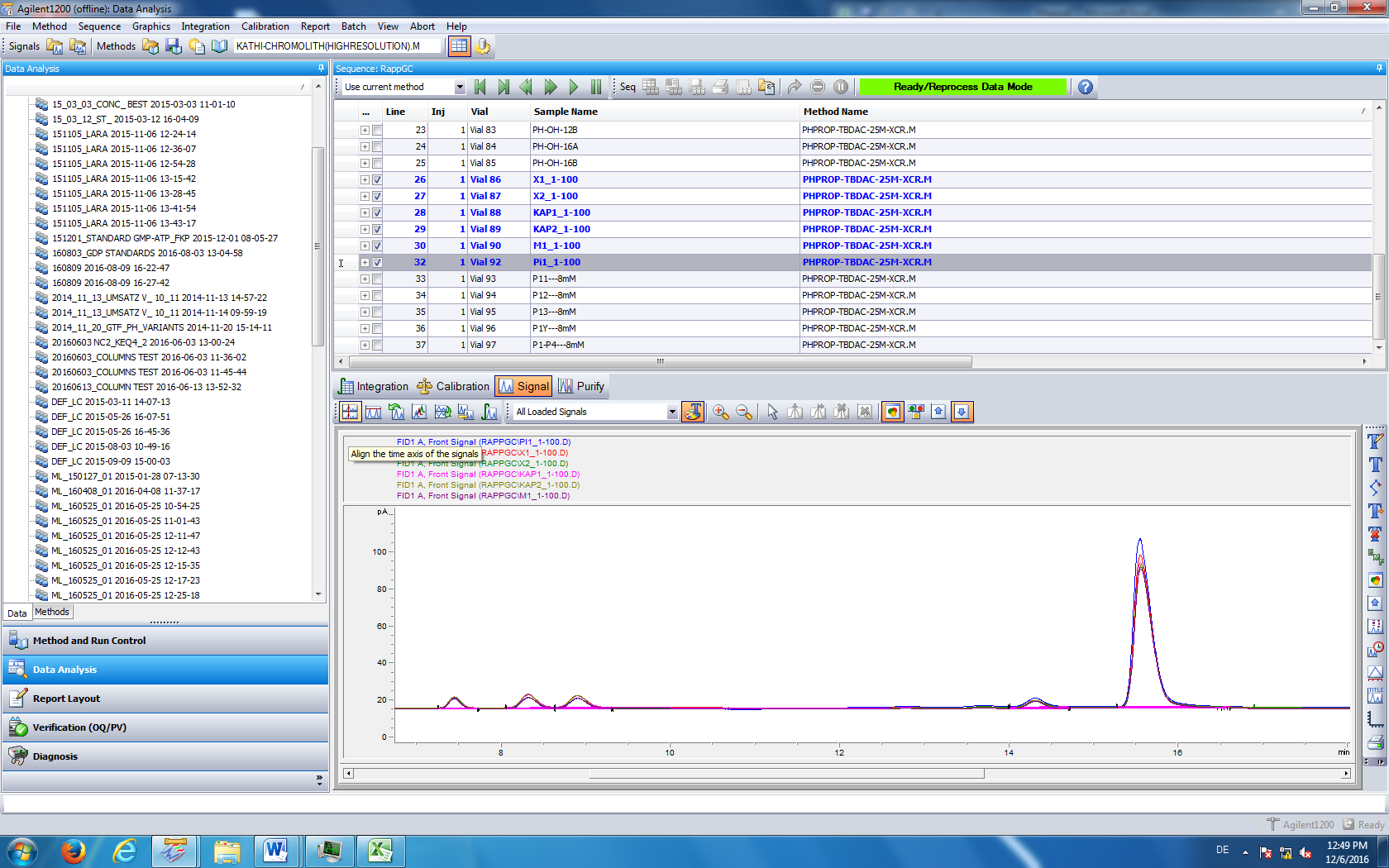**  *(R)-*2-phenylpropanol  *rac*-2-phenylpropanal  Acetophenone |
| --- |

*Figure S4*. GC traces for bioreduction replicates (N=6) of 1M 2-phenylpropanal reacted with 40g_CDW_/L and 6mM NAD^+^.

*Table S3 (data corresponding to Figure S4)*. Retention times for main products and by-products on chiral GC.

| Analyte | Retention time (min) |
| --- | --- |
| (*R*)-2-Phenylpropanal | 8.2 |
| (*S*)-2-Phenylpropanal | 8.8 |
| (*R*)-2-Phenylpropanol | 14.8 |
| (*S*)-2-Phenylpropanol | 15.4 |
| Acetophenone | 6.9 |

**6. ^1^H-NMR**

**Bioreduction analysis**


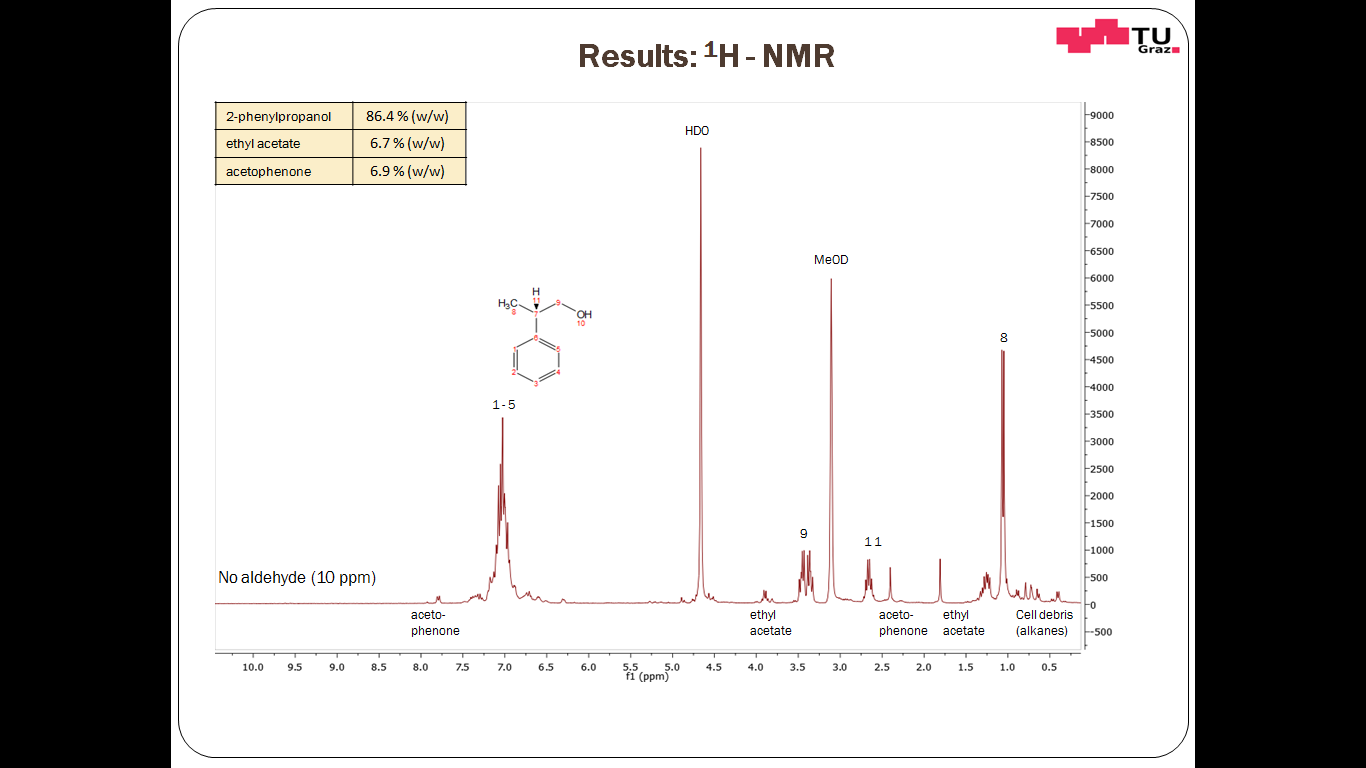
The isolated compounds (extraction with ethyl acetate) from a bioreduction mixture of 1M 2-phenylpropanal reacted with 40g_CDW_/L and 6mM NAD^+^ were analyzed by ^1^H-NMR.

*Figure S5*. ^1^H-spectra in MeOD. The graph shows a spectrum of isolated compounds from a reaction with 78 % analytical yield (HPLC). Spectra were recorded immediately after work-up. Signals assigned to ethyl acetate are found at 1.75 and 3.9ppm, a signal assigned to acetophenone is found at 2.45ppm.
